# Community Perception and Client Satisfaction about the Primary Health Care Services in an Urban Resettlement Colony of New Delhi

**DOI:** 10.4103/0970-0218.43232

**Published:** 2008-10

**Authors:** Binod Kumar Patro, Rakesh Kumar, Anil Goswami, Baridalyne Nongkynrih, Chandrakant S Pandav, UG Study Group

**Affiliations:** Centre for Community Medicine, All India Institute of Medical Sciences, New Delhi - 110 029, India

**Keywords:** Client satisfaction, community perception, India, primary healthcare

## Abstract

**Research Question:**

This study attempts to assess the community perception and client satisfaction of primary health care services provided by mobile health clinics.

**Objectives:**

To assess the awareness of the community about mobile health clinic services and its utilization in an urban area and to assess the client satisfaction of those who have utilized the services.

**Study Design:**

A cross-sectional community-based study.

**Setting:**

Dr. Ambedkar Nagar, urban resettlement colony of New Delhi.

**Study Period:**

July 2006 and September 2006.

**Participants:**

For exit interviews, patients who have utilized the mobile health clinic; for community interviews, an adult member present in the family.

**Materials and Methods:**

For the community survey, two blocks were randomly chosen and the interview was carried out by house visits. For exit interviews, patients were selected randomly from those attending the mobile health clinic.

**Statistical Analysis:**

Descriptive statistical analysis.

**Results and Conclusions:**

A total of 377 interviews were conducted (295 community interviews and 82 exit interviews). It was seen that 82% were aware of the mobile health clinic but more than two-thirds preferred private practitioners; reasons given were that they have more trust in private practitioners, convenient timings, and less waiting time. Approximately two-thirds to three-fourths of the clients were satisfied with the mobile health clinic services. Client satisfaction is an important measure of the quality of health care and needs to be addressed in order to improve the utilization of primary health care services in urban areas.

## Introduction

The Alma-Ata international conference defined Primary Health Care as “the essential health care made universally accessible to individuals and acceptable to them, through their full participation and at a cost the community and country can afford.”(1) The Government of India was a signatory to Alma-Ata's declaration and committed to providing quality primary health care services to achieve Health for All by the year 2000. Community participation is one of the key principles on which primary health care is established—others being equitable distribution, inter-sectoral coordination, and appropriate technology. In the way of providing primary health care services, the Government of India has made impressive growth in terms of the establishment of primary health care institutions across rural, tribal hard-to-reach sectors. However, shortcomings in the delivery of primary health care services has resulted in lesser utilization rates and more dependence on private health care service providers.(2) The delivery of health services for the poor people in urban areas and disadvantaged population remains a challenge in India.

Information about community perception with a thorough understanding of the needs and expectations of the community about the health care services can help in better delivery and higher utilization of health services. This study attempts to assess the community perception and client satisfaction of primary health care services provided by mobile health clinics with the following objectives:

- To assess the awareness of the community about mobile health clinic services and its utilization in an urban area.
- To assess the client satisfaction based on those people who have utilized the services.

## Materials and Methods

The Centre for Community Medicine at the All India Institute of Medical Services is actively involved in research and development in the area of primary health care. As a part of service delivery, the center provides primary health care services to the urban resettlement colony of New Delhi. Six administrative blocks of Dr. Ambedkar Nagar, situated in South Delhi, are provided ambulatory primary health care services through a structured urban health program since the year 2001. Curative health services are provided through a mobile health clinic 5 days a week wherein treatment for minor ailments is provided and specialized clinics (antenatal clinic and immunization clinic) are provided twice a week. Preventive and promotive services are provided at the household level through a team of health workers, a public health nurse, and medical social service officers.

A community-based cross-sectional survey was conducted between July 2006 and September 2006. Keeping feasibility and logistics in mind, it was decided to include 300 community interviews and 100 exit interviews. For the community survey, two blocks were selected randomly for inclusion into the study. Households were considered as a unit of study. The informant was the head of the household or any individual aged 15 years or older who was present during the time of the study. Verbal informed consent was obtained form the study participants. Alternate households were chosen for inclusion in the study. For exit interviews, patients were selected randomly from those who attended the mobile health clinic during the study period. Care was taken to include all days of the week and also to include special activities like immunization and antenatal clinics.

Interview schedules were developed for exit interviews and community surveys separately with inputs from the existing literature. Different domains of the interview schedule were demographic schedule, awareness and utilization, level of satisfaction, and suggestions and expectations. The interview schedule was prepared in English and translated into Hindi (local dialect). Pre-testing of the study instrument was done in an independent area from the study site and relevant modifications were made. Operational definitions were defined for awareness, utilization, waiting time, consultation time, and time for procurement of drugs. The level of satisfaction was assessed in two stages; first satisfied or not satisfied, then extremely or moderately in both categories.

## Results

A total of 377 interviews were conducted, which included 295 community interviews and 82 exit interviews. The refusal rate was approximately 4% in both categories of respondents. In the community surveys, around two-thirds of the respondents were female, three-fourths were in the age group of 20 to 59 years old, and four-fifths were married, most with either primary or no education. In the exit interviews, the demographic distribution of the respondents was similar with most of them being females in the age group of 20 to 59 years old with either primary or no education [Table 1].

**Table 1:** Demographic details of study respondents

Variables	Community surveys (%) n = 295	Exit interviews (%) n = 82
Sex		
Male	105 (35.6)	18 (22)
Female	190 (64.4)	64 (78)*
Age group (years)		
<20	45 (15)	03 (3.6)
20–59	231 (78)	75 (91.5)
≥60	19 (7)	04 (4.9)*
Marital status		
Married	243 (82)	81(98.7)
Unmarried	52 (18)	01(1.3)**
Education		
Illiterate	100 (34)	33 (40)
Primary	133 (45)	34 (41.5)
Secondary	29 (9.8)	05 (6)
Higher secondary	12 (4)	03 (3.8)
Graduate and above	21 (7.2)	07 (8.7)*

* *P* value > 0.05 = not significant

** *P* value < 0.05 = significant

### Treatment preference

In the community surveys, more than two-thirds (70.5%) of the respondents listed private practitioners/private hospitals as their preferred choice of health care facility in case of illness followed by the mobile health clinic (12.9%). Similarly, in exit interviews that were taken after the respondents had availed of the services of the mobile health clinic, about half (48.8%) of them preferred the services of a private practitioner/private hospital as a first choice followed by the mobile health clinic (35.3%).

### Awareness and utilization of mobile health clinics

Awareness about the services provided by mobile health clinics was assessed only in the community survey. About 82% of the respondents were aware of the mobile health clinic. Despite this high awareness, only just over half (54.9%) of those who were aware of the mobile health clinic had ever utilized the services. Some of the major reasons for non utilization of services were more faith in private practitioners, inconvenient timing of the mobile health clinic, long queues, non availability of all drugs, and investigations.

### Waiting time and consultation time

Waiting time was recorded as reported by the respondents and captured in two categories. In addition to the waiting time, a record was made about the duration of consultation with the physician. First, waiting for consultation was defined as the time from registration to meeting with the physicians. Secondly, waiting time to procure drugs was defined as time from consultancy with the physician to procurement of drugs from the pharmacy. The average waiting time reported in both the community surveys and exit interviews was approximately 30 to 35 minutes. The consultation time was approximately 5 minutes. The waiting time for getting the drugs from the pharmacy after consultation with the physician was about 15 to 20 minutes. The ranges of waiting time and consultation time are presented in Table 2.

**Table 2:** Waiting and consultation time

Waiting time for consultancy (minutes)	Community surveys (%)	Exit interviews (%)
<15	29 (21.8)	9 (11)
15–30	32 (24)	34 (41.4)
30–60	53 (40)	23 (28)
>60	19 (14.2)	16 (19.6)
**Waiting time for drugs (minutes)**		
<10	45 (33.8)	36 (44)
10–15	27 (20.3)	19 (23)
15–30	34 (25.6)	15 (18.3)
>30	27 (20.3)	12 (14.7)
**Consultation time (minutes)**		
<5	65 (48.9)	47 (57.3)
5–10	46 (34.6)	32 (39)
10–15	16 (12)	3 (3.7)
>15	6 (4.5)	0

### Level of satisfaction

The level of satisfaction was assessed in two stages. In the first stage, respondents were asked whether they were satisfied or dissatisfied with various aspects of the health services provided by the mobile health clinic. Further, they were asked to categorize their response into satisfied or very satisfied and dissatisfied or very dissatisfied, respectively into both the groups. The level of satisfaction was assessed across all the components of primary health care services using 12 items.

### Level of satisfaction in the community surveys

The domains where the satisfaction level was rated as very high included immunization services, competency of the doctor/health staff, and behavior of the doctor/health staff. There was a high level of dissatisfaction with the availability of medicines and the availability of investigations. Around one-third of the respondents were dissatisfied with the timings kept by the clinic while two-fifths of the respondents were dissatisfied with the waiting time [Table 3].

**Table 3:** Level of satisfaction in the community interviews

Level of satisfaction	Very satisfied (%)	Satisfied (%)	Very dissatisfied (%)	Dissatisfied (%)
Distance from home	32 (24)	55 (41.3)	22 (16.6)	24 (18.1)
Timings kept by the clinic	42 (31.5)	44 (33)	25 (18.9)	22 (16.6)
Waiting time	56 (42.1)	21 (15.8)	25 (18.9)	31 (23.2)
Consultation time	22 (16.6)	55 (41.3)	30 (22.6)	26 (19.5)
Behavior of doctor/health staff	22 (16.6)	81 (61)	23 (17.2)	7 (5.2)
Competence of doctor/health staff	21 (15.8)	84 (63.1)	23 (17.2)	5 (3.9)
Health information provision	28 (21)	73 (54.9)	20 (15.1)	12 (9)
Physical examination	23 (17.2)	65 (49)	23 (17.2)	22 (16.6)
Availability of medicines	29 (21.8)	43 (32.4)	30 (22.6)	31 (23.2)
Availability of investigations	17 (21.5)	21 (26.6)	26 (32.9)	15 (19)
Immunization services	7 (11.9)	44 (74.6)	5 (8.4)	3 (5.1)
Relief of symptoms	28 (26)	43 (39.9)	30 (27.6)	7 (6.5)

### Level of satisfaction among exit interviews

In the exit interviews, the level of satisfaction with the health services was quite high. The behavior of doctors/health staff and immunization services were two domains where the level of satisfaction was quite high. As in the community surveys, waiting time and availability of medicines and investigations were the domains with some level of dissatisfaction, albeit the level of dissatisfaction was less. In contrast to the community surveys, very few respondents were dissatisfied with the timings kept by the clinic [Table 4].

**Table 4:** Level of satisfaction in the exit interviews

Level of satisfaction	Satisfied (%)	Very satisfied (%)	Dissatisfied (%)	Very dissatisfied (%)
Distance from home	35 (42.7)	36 (43.9)	9 (11)	2 (2.4)
Timing of clinic	32 (39.1)	46 (56)	4 (4.9)	0
Waiting time	26 (31.7)	31 (37.8)	23 (28)	2 (2.5)
Consultation time	42 (51.2)	27 (33)	8 (9.8)	5 (6)
Behavior of doctor/health staff	53 (64.6)	29 (35.4)	0	0
Competence of doctor/health staff	47 (57.5)	25 (30.5)	5 (6)	5 (6)
Health information provision	32 (39.1)	26 (31.7)	17 (20.7)	7 (8.5)
Physical examination	23 (28)	36 (43.9)	17 (20.7)	6 (7.4)
Availability of medicines	36 (43.9)	16 (19.5)	26 (31.7)	4 (4.9)
Availability of investigations	26 (31.7)	26 (31.7)	18 (22)	12 (14.6)
Immunization services	26 (63.4)	10 (24.4)	4 (9.8)	1 (2.4)
Relief of symptoms	22 (42.3)	27 (52)	2 (3.8)	1 (1.9)

## Discussion

An increased emphasis on client satisfaction is driven by the perceived need for the democratization of primary health care. Patient satisfaction as a measure of health care is an important outcome measure. It is useful in assessing consultations and patterns of communications. If used systematically, feedback enables a choice between alternatives in organizing or providing health care.(3)

The efficacy of medical treatment is enhanced by greater patient satisfaction. It can also be taken as the proxy measure for the quality of health care. Our study is restricted to the views of the user's of the health services and it identifies various impediments in the delivery of health care services that may be important to the users of the healthcare services but may appear trivial to healthcare personnel. Incorporating the views of the users in the management of the health services will lead to fewer unsatisfied users.

This study attempts to assess client satisfaction in two distinct settings: community surveys and exit interviews. Though these two settings are inherently different, they point towards the same trend. Most of the respondents were females, which can be explained by the fact that the interviews were conducted during working hours and most of the males were away at work. Most of the respondents were either illiterate or educated up to the primary level. These factors could have affected the level of satisfaction; because of illiteracy and ignorance, the awareness and accessibility about other health facilities is limited among the respondents; therefore they probably would not have other options with which to compare. A study conducted in Egypt reported that the level of patient satisfaction with primary health care services is not affected by gender and educational level;(4) however, in this setting, it may not be applicable.

In community surveys, though the awareness about the mobile health clinic was quite high, only about half of the respondents who were aware had ever utilized the services of the mobile health clinic. The reasons cited for the non utilization were more faith in private practitioners, inconvenient timing of the mobile health clinic, long queues, unavailability of drugs and investigations, and the distance from homes. More faith in private practitioners could be due to ready availability in case of need as the mobile health clinic runs for only 2 hours in the morning.

The mean waiting time was similar in the community surveys and in exit interviews, which was about 30 minutes. The waiting time correlates well with the average waiting time in similar settings.(5,6) It also emerges as one of the major areas of dissatisfaction with the health services as well as the cause of non utilization. A reduction in the waiting time could improve patient satisfaction and enhance the utilization of health services provided by the mobile health clinic. The average consultation time in community surveys was more than 5 minutes while it was reported to be less than 5 minutes in exit interviews. However, this was twice as high as that reported from another study in similar settings.(6) This could be the explanation for the higher level of satisfaction with the physician-related domains *vs.* the behavior of doctors/health staffs, competence of doctors, provision of information, and physical examination.

Non availability of certain drugs and investigations emerged as major areas of client dissatisfaction. This correlates with the findings of the other studies.(4–7) This is due to the general perception that being a government organization, the mobile health clinic should provide all the medicines and investigations free of cost.

Data collection was done by undergraduate medical students, which may bias the response of the participants but it is less likely to be so as in the community surveys patients were not seeking the services of the mobile health clinic at the time of the interview.

## Limitations

Data collection was carried out by the members of the mobile health team, which might have introduced a bias. Secondly, the waiting time and consultation time were self-reported so recall bias might have been introduced.

## Conclusion

One of the measures of the quality of health care is by assessing client satisfaction. In this study, we have attempted to assess the level of satisfaction of the users of the mobile clinic and the reasons for non utilization. In India, there is a gross disparity in the distribution of health services in urban areas. While there are multiple agencies that provide health care in urban areas, there is inequity in the way the services are distributed. The urban poor still need to depend on government services for basic health care. Therefore, as we are providing facilities for preventive and curative health care delivered at the doorstep of the people through the mobile health clinic, it is important to ascertain the level of utilization and reasons for non utilization. These have to be addressed and looked into in order to improve utilization. In resource-constrained set-ups like in all developing countries, all efforts should be undertaken to bring about the maximum efficiency of health care delivery.

## References

[1] (1978). Primary Health Care, HFA Sr No. 1.

[2] (1995-96). Utilization and expenditure of Health Services in India 52^nd^ Round. National Sample Survey Organization, Govt of India.

[3] Fitzpatrick R (1991). Surveys of patient satisfaction: Important general considerations. BMJ.

[4] Gadallah M, Zaki B, Rady M, Anwer W, Sallam I (2003). Patient satisfaction with primary health care services in two districts of lower and upper Egypt. East Mediterr Health J.

[5] Ortola P, Blanquer JJ, Rodriquez JJ, Rodrigo O, Villagrasa F, Climent JA (1993). User satisfaction in primary care: Result of a home survey. Aten Primaria.

[6] Mendoza Aldana J, Piechulek H, al-Sabir A (2001). Client satisfaction and quality of health care in rural Bangladesh. Bull World Health Organ.

[7] Baltussen RM, Yé Y, Haddad S, Sauerborn RS (2002). Perceived quality of care of primary health care services in Burkina Faso. Health Policy Plan.

